# AGTR1rs5186 Polymorphism Is Associated with the Risk of Restenosis after Percutaneous Coronary Intervention: A Meta-Analysis

**DOI:** 10.3390/jcdd9110406

**Published:** 2022-11-21

**Authors:** Feng Lv, Yufeng Jiang, Yebao Wang, Ting Zhang, Yafeng Zhou

**Affiliations:** 1 Department of Cardiology, Suzhou Dushu Lake Hospital, Dushu Lake Hospital Affiliated to Soochow University, Medical Center of Soochow University, Suzhou 215125, China; 2 Department of Cardiology, Shengzhou People’s Hospital, The First Affiliated Hospital of Zhejiang University Shengzhou Branch, Shengzhou 312400, China

**Keywords:** cardiovascular disease, rs5186, percutaneous coronary intervention, polymorphism

## Abstract

Background: Progress has been made in genetic investigations on restenosis for the past 20 years, many studies regarding *AGTR1* rs5186 polymorphism and restenosis after percutaneous coronary intervention (PCI) have been published, but the result remains controversial. The study aimed to explore the relationship between rs5186 polymorphism and the risk of restenosis after PCI. Methods: We performed a systematic search on PubMed, Web of Science, Embase, CNKI, and Wan Fang databases up to December 2021. Two authors individually extracted all useful data of each study involved in this meta-analysis and assessed the study quality using the Newcastle-Ottawa scale. Odds ratios (ORs) and 95% confidence intervals (CIs) were combined in different genetic models for evaluation using a random-effects model or fixed-effect model. Results: There were eventually 8 studies of 1111 cases and 4097 controls eligible for this meta-analysis. Significant associations were found between rs5186 polymorphism and restenosis after PCI.allelic (OR: 1.31, 95% CI: 1.17–1.47, *p* < 0.001), homozygous (OR: 1.90, 95% CI: 1.50–2.44, *p* < 0.001), heterozygous (OR: 1.10, 95% CI: 0.93–1.29, *p* = 0.27), recessive (OR: 1.80, 95% CI: 1.37–2.36, *p* < 0.001), dominant genetic model (OR: 1.24, 95% CI: 1.06–1.44, *p* = 0.006). Subgroup analyses indicated a significant association in Asians. Conclusions: The rs5186 polymorphism in the *AGTR1* gene increases the risk of restenosis after PCI in Asians significantly.

## 1. Introduction

Cardiovascular disease (CVD) remains a major cause of premature mortality and rising healthcare costs [1]. The CVD burden continues its decades-long rise in almost all countries outside high-income countries, and alarmingly, the age-standardized rate of CVD has begun to rise in some locations where it was previously declining in high-income countries [2]. There is an urgent need to implement interventions.

Coronary atherosclerotic heart disease (CHD) is one of the major cardiovascular diseases. In addition to medical therapy, percutaneous coronary intervention (PCI) has become an important treatment for CHD. Primary PCI modalities include percutaneous transluminal coronary balloon angioplasty, coronary stenting, and others, which reduce CHD-related mortality significantly [3]. However, the disadvantages of coronary restenosis after PCI have become increasingly obvious. Although the rate of restenosis has decreased in recent years with second-generation drug-eluting stents, restenosis after PCI still affects patient outcomes [4,5,6]. It has been well-reported that age [7], hypertension [8], diabetes [9], and dyslipidemia [10] are risk factors for restenosis. However, coronary lesion-specific interventional procedures can also contribute to restenosis [11].

The pathophysiological of restenosis after PCI is damage to vascular endothelial cells, leading to hyperplasia of the neovascular endothelium. Angiotensin II in the renal angiotensin aldosterone system (RAAS) would stimulate the expression of smooth muscle cell growth factor to promote smooth muscle cell hyperplasia, migration, and thus the occurrence of restenosis [12]. With the rapid development of sequencing technology, much progress has been made in genetic investigations on restenosis. For the past 20 years, many case-control-designed studies [13,14,15,16,17,18,19,20] regarding the Ang II type 1 receptor (*AGTR1*) rs5186 polymorphism and restenosis after PCI have been published. Due to the small sample size and low statistical power of individual studies, the consolidated result is still controversial. In this paper, we conducted a meta-analysis of the rs5186 gene polymorphism in all patients with restenosis versus no after PCI from the enrolled studies to investigate the association between rs5186 polymorphism and the risk of restenosis after PCI.

## 2. Materials and Methods

We followed the methods of Jiang et al. [21] to perform our meta-analysis. Since this meta-analysis was based on published studies, patient consent and ethical approval were not required.

### 2.1. Search Strategy

A systematic search was conducted in the PubMed, Web of Science, Embase, CNKI, and Wan Fang databases from inception to December 2021 with no language restrictions, combined with a manual search of reference lists from the identified articles. The following terms were used in the literature search: restenosis, rs5186, *AGTR1*, A1166, polymorphism, variant, and mutation. This meta-analysis was performed according to the Preferred Reporting Items for Systematic Reviews and Meta-Analyses (PRISMA) [22]. The protocol for this systematic review was registered on INPLASY (International Platform of Registered Systematic Review and Meta-analysis Protocols) (Unique ID number) and is available in full on inplasy.com (ID: INPLASY2022110054).

### 2.2. Selection and Exclusion Criteria

Studies meeting the following criteria were included: (1)Case-control studies; (2) Investigating the association of the rs5186 polymorphism and risk of restenosis; (3)Studies had data of odds ratios (ORs) and 95% confidence intervals (95% CI) or had sufficient data to calculate it.

### 2.3. Data Extraction

Two of the authors independently extracted data from eligible studies. The process was as follows: (1) read the article title and exclude duplicate studies; (2) Titles and abstracts were read, and irrelevant literature was excluded; (3) The full texts were read in detail, and the literature that met the inclusion and exclusion criteria were finally identified. Conflicts were discussed with a third reviewer. Extraction of study data: author, publication year, country, ethnicity, number of patients, source of controls, PCI type, genotyping method, and genotype distribution. The 9-point Newcastle-Ottawa Scale (NOS) was used to assess the methodological quality included [23].

### 2.4. Statistical Analysis

This meta-analysis was performed using Stata version 14.0. We did Hardy-Weinberg equilibrium (HWE) tests for every study included. We explored the associations between rs5186 polymorphism and risk of restenosis by combining ORs and 95% CIs under a random-effect or fixed model. A random-effects model for pooled analysis would be adopted when I^2^ >50% indicating heterogeneity. Otherwise, the fixed-effect model would be used. We also performed subgroup analyses to identify the underlying heterogeneity according to ethnicity, study sample size, and PCI type. The analyses were conducted in 5 genetic models: allele (A allele distribution frequency of rs5186 polymorphism), homozygote model (AA vs. CC), heterozygote model (AC vs. AA), recessive model (CC vs. AC + AA) and dominant model (CC + CC vs. AA). A sensitivity analysis was performed to evaluate the stability of the results. We investigated publication bias by calculating the Egger test and drawing the Begg funnel plot.

## 3. Results

### 3.1. Study Characteristics

**Figure 1:**
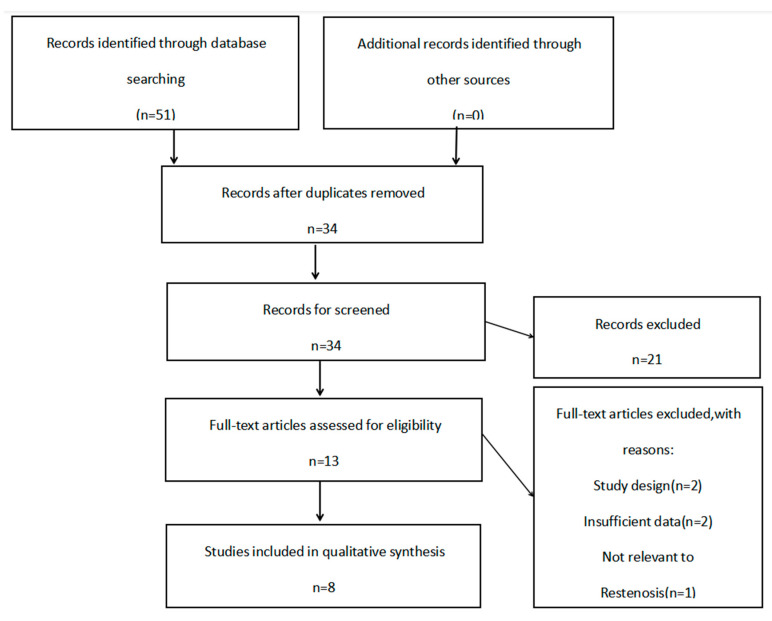
The PRISMA flow diagram of the study selection and exclusion.

The search of the five databases identified 51 records. After removing the duplicated studies, there were 34 studies left for screening. Twenty-one irrelevant pieces of literature were excluded after reading the title and abstract of the article. Finally, 13 studies were read in full-text, and five full-text articles were excluded because of unmatched study design (*n* = 2), insufficient data (*n* = 2), and not relevant to restenosis (*n* = 1). Figure 1 shows the complete procedure of study selection and exclusion. There were eventually 8 studies [13,14,15,16,17,18,19,20] of 1111 cases and 4097 controls eligible for this meta-analysis. Characteristics of the studies included in the meta-analysis are shown in Table 1. All articles were published in English. The sample sizes ranged from 111 to 2946 of all eligible studies. The race of the included studies were Asian (China, *n* = 3) and Caucasian (France, Germany, Russia, The Netherlands, *n* = 5). All included studies fitted in with the HWE test. The results of NOS are shown in Table 2. The NOS of all eligible studies in our meta-analysis was >6 points, representing a good study quality. Genotype distribution and allele frequency in cases and controls of each study are shown in Table 3.

### 3.2. Quantitative Synthesis

**Figure 2:**
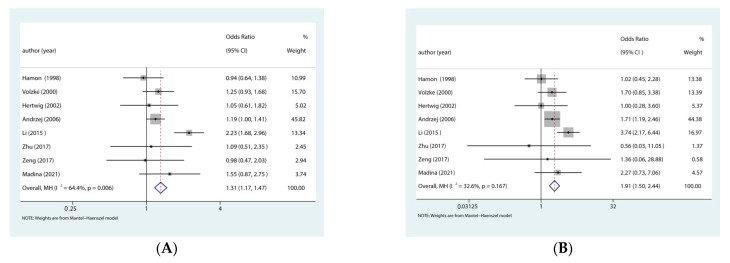

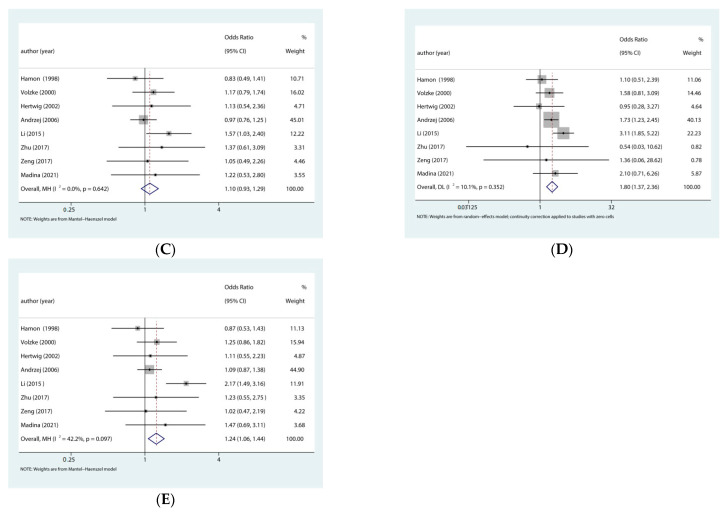
Forest plot from the meta-analysis on the association of the rs5186 polymorphism and risk of restenosis after PCI. (**A**) allele model: C vs. A; (**B**) homozygote model:CC vs. AA; (**C**) heterozygote model: AC vs. AA; (**D**) recessive model: CC vs. AC + AA; and (**E**) dominant model: CC + AC vs. AA. CI = confidence interval, OR = odds ratio [13,14,15,16,17,18,19,20].

The meta-analysis indicated a significant association between *AGTR1* rs5186 polymorphism and restenosis after PCI.allelic (OR: 1.31, 95% CI: 1.17–1.47, *p* < 0.001), homozygous (OR: 1.90, 95% CI: 1.50–2.44, *p* < 0.001), heterozygous (OR: 1.10, 95% CI: 0.93–1.29, *p* = 0.27), recessive (OR: 1.80, 95% CI: 1.37–2.36, *p* < 0.001), dominant genetic model (OR: 1.24, 95% CI: 1.06–1.44, *p* = 0.006) in the whole population (Figure 2).

**Figure 3:**
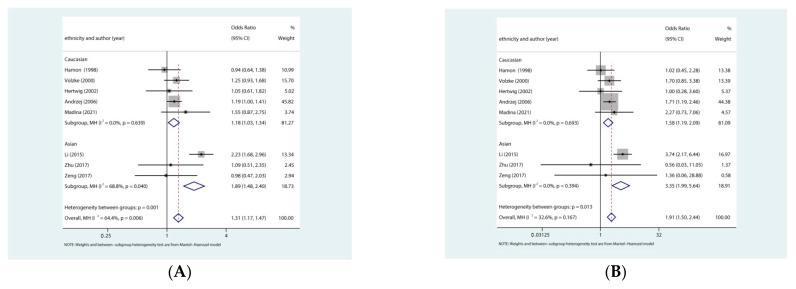

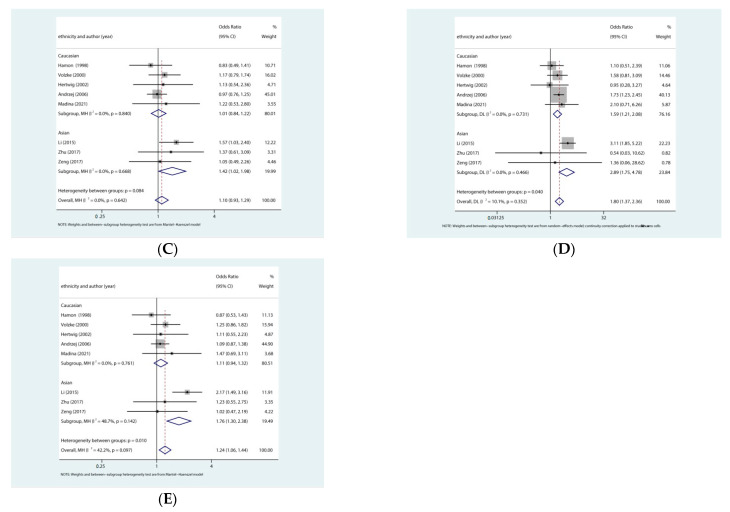
Subgroup meta-analysis by ethnicity of the association of the rs5186 polymorphism and risk of restenosis after PCI. (**A**) allele model: C vs. A; (**B**) homozygote model:CC vs. AA; (**C**) heterozygote model: AC vs. AA; (**D**) recessive model: CC vs. AC + AA; and (**E**) dominant model: CC + AC vs. AA. CI = confidence interval, OR = odds ratio [13,14,15,16,17,18,19,20].

In the subgroup analyses by ethnicity (Figure 3), the association grew stronger with higher ORs in Asian under all genetic models: allelic (OR: 1.89, 95% CI: 1.48–2.40, *p* < 0.001), homozygous (OR: 3.35, 95% CI: 1.99–5.64, *p* <0.001), heterozygous (OR: 1.42, 95% CI: 1.02–1.98, *p* = 0.04), recessive (OR: 2.89, 95% CI: 1.75–4.78, *p* <0.001), dominant genetic model (OR: 1.76, 95% CI: 1.30–2.38, *p* < 0.001). In the Caucasian subgroup, we also found association under allelic (OR: 1.18, 95% CI: 1.03–1.34, *p* = 0.02), homozygous (OR: 1.58, 95% CI: 1.19–2.09, *p* = 0.002) recessive (OR: 1.59,95% CI: 1.21–2.09, *p* = 0.01). In summary, our meta-analysis suggested that rs5186 polymorphism in the *AGTR1* gene increased the risk of restenosis after PCI, particularly in Asians. We also carried out subgroup analyses according to sample size, and PCI type. The detailed information is presented in Table 4. A similar association was observed in both sample size (≥400) and PCI type (stent) subgroups that rs5186 polymorphisms in the *AGTR1* gene increased the risk of restenosis after PCI.

### 3.3. Sensitivity Analysis

**Figure 4:**
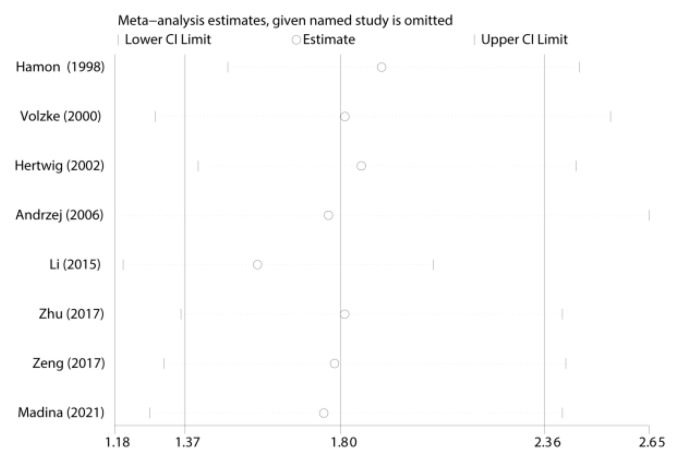
Sensitivity analysis of the pooled OR coefficients on the relationship between rs5186 polymorphism and risk of restenosis after PCI.CI = confidence interval, OR = odds ratio [13,14,15,16,17,18,19,20].

We carried out the sensitivity analysis to find whether the omission of each study would change the pooled ORs quantitatively. No changed results are shown after the individual study was omitted in Figure 4, which supplied evidence to prove the increased risk of the rs5186 polymorphism to restenosis after PCI (Figure 4).

### 3.4. Publication Bias

**Figure 5:**
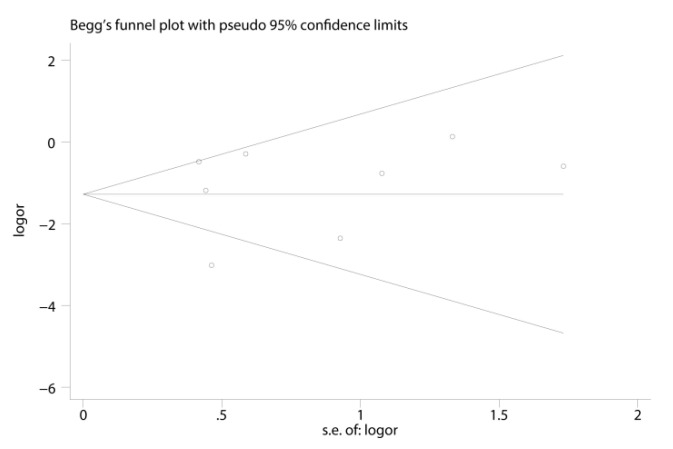
Begg’s funnel plot with pseudo 95% confidence limits.

We computed the Egger test and drew the Begg funnel plot to estimate the publication bias. We could see that all eight studies were distributed on two sides of the Begg funnel plot (Figure 5), which implied no publication bias in our meta-analysis (Egger test: *p* = 0.59). 

## 4. Discussion

CVD is the leading cause of global mortality. Prevalent cases of total CVD nearly doubled from 271 million in 1990 to 523 million in 2019, and the number of CVD deaths steadily increased from 12.1 million in 1990, reaching 18.6 million in 2019 [2]. CHD, as one of the major CVD, has received more attention than before. Despite great progress in reducing CHD deaths over the past decades. Percutaneous coronary intervention (PCI) is an alternative to revascularization in patients with CAD. Although intracoronary stent implantation has been shown to reduce coronary vessel restenosis significantly, restenosis problems remain even after drug-eluting stents become one of the most promising techniques in the current field of interventional cardiology. Mechanisms leading to restenosis after balloon angioplasty include elastic recoil and vascular remodeling. Excessive neointima formation, a process resulting from smooth muscle cell proliferation and extracellular matrix synthesis, is also a major cause of luminal restenosis after PCI.

The renal angiotensin aldosterone system (RAAS) has been implicated in the development and progression of neointimal hyperplasia predominantly through angiotensin II(Ang II) [24] Angiotensin-converting enzyme converts angiotensin I (Ang I) to Ang II which promotes the migration and proliferation of VSMCs, causes vasoconstriction and regulates expression of adhesion molecules via its major cellular receptor, *AGTR1*. Genes regulating levels and activity of the RAAS contribute to vasoconstriction and neointimal hyperplasia and are associated with restenosis following intracoronary stent placement [16,25]. With the rapid development of sequencing technology, much progress has been made in the genetic investigation of restenosis. Many studies [13,14,15,16,17,18,19,20] found that the rs5186 polymorphism of *AGTR1* was a risk factor for restenosis after PCI [18].

Our meta-analysis consolidated eight eligible studies on the rs5186 polymorphism of *AGTR1* and the relationship with restenosis after PCI. All results indicated that the rs5186 polymorphism would increase the risk of restenosis after PCI. Subgroup analyses showed a higher risk of restenosis after PCI in subjects with the risk allele in the Asian population; we also conducted subgroup analyses according to sample size and PCI type. A similar association was observed in both sample size (≥400) and PCI type (stent) subgroups that rs5186 polymorphism in the *AGTR1* gene increase the risk of restenosis after PCI. There was no publication bias in our meta-analysis.

Limitations of our meta-analysis should be acknowledged. First, all eight studies included in this meta-analysis were written in English and Chinese, so studies in other languages and possibly unpublished articles did not attend, which may cause selection bias. Second, there were no studies including Africans. The sample size of three studies, including Asians, was less than five studies, including Caucasians in this meta-analysis, which may influence the results. Third, genetic susceptibility may also depend on the coincidence of several gene polymorphisms acting together, which may influence the results.

We finally concluded that the rs5186 polymorphism in the *AGTR1* gene increases the risk of restenosis after PCI in Asians. It could be a promising locus for genetic therapy in the clinical management of restenosis after PCI. More case-control studies need to be carried out to further validate and strengthen the conclusion of this meta-analysis.

## Figures and Tables

**Table 1:** Characteristics of the studies included for meta-analysis.

Author	Year	Country	Ethnicity	Age (Years)		Gender (M/F)		Comorbidities	Source of Controls	Genotyping Method	Polymorphism	NOS Score	HWE Test
				Case	Control	Case	Control						
Hamon et al. [13]	1998	France	Caucasian	NR		229/42		HTN, diabetes, CAD	HB	PCR	rs5186	7	0.35
Volzke et al. [14]	2000	Germany	Caucasian	59.9 (8.9)	60.6 (8.6)	126/34	262/89	HTN, diabetes, CAD	HB	PCR–RFLP	rs5186	8	0.96
Hertwig et al. [15]	2002	Germany	Caucasian	59.7 (7.9)	58.7 (9.2)	37/9	80/19	HTN, diabetes, CAD	HB	PCR–RFLP	rs5186	7	0.60
Wijpkema et al. [16]	2006	The Netherlands	Caucasian	62 (11)		2121/825		HTN, diabetes, CAD	HB	PCR–RFLP	rs5186	8	0.12
Li et al. [17]	2015	China	Asian	72.2 (4.2)	72.2 (4.1)	138/169	85/91	HTN, diabetes, CAD	HB	PCR–RFLP	rs5186	6	0.04
Zhu et al. [18]	2017	China	Asian	65.3 (11.5)	63.7 (11.6)	59/16	205/72	HTN, diabetes, CAD	HB	PCR	rs5186	7	0.36
Zeng et al. [19]	2017	China	Asian	61.3 (6)	60.5 (6.15)	41/13	273/98	HTN, diabetes, CAD	HB	PCR	rs5186	8	0.65
Azova et al. [20]	2021	Russian	Caucasian	60 (10.1)	58.8 (8)	94/19		HTN, diabetes, CAD	HB	PCR–RFLP	rs5186	7	0.20

Case-control design was used in all included studies. PCR-RFLP = polymerase chain reaction restriction fragment length polymorphism; year = publication year; NOS = Newcastle-Ottawa scale; HWE = Hardy–Weinberg equilibrium; HB = hospital-based; HTN = hypertension; CAD = coronary artery diseaseCase-control design was used in all included studies. PCR-RFLP = polymerase chain reaction restriction fragment length polymorphism; year = publication year; NOS = Newcastle-Ottawa scale; HWE = Hardy–Weinberg equilibrium; HB = hospital-based; HTN = hypertension; CAD = coronary artery disease.

**Table 2:** The results of Newcastle-Ottawa Scale.

Author	Selection	Comparability	Exposure
Hamon et al. [13]	***	**	**
Volzke et al. [14]	***	**	***
Hertwig et al. [15]	***	**	**
Wijpkema et al. [16]	***	**	***
Li et al. [17]	**	**	**
Zhu et al. [18]	***	**	**
Zeng et al. [19]	***	**	***
Azova et al. [20]	***	**	**

The quality of each study was assessed based on a 9-point Newcastle-Ottawa scale. The maximum points for selection, comparability and exposure in the table, (4 points for selection, 2 points for comparability, and 3 points for exposure). Each * is 1 point in the table. All eligible studies in our meta-analysis were >6 points.

**Table 3:** ANT1R rs5186 polymorphism genotype distribution and allele frequency in cases and controls.

	Genotype (N)								Allele Frequency (N, %)					
	Cases				Controls				Cases			Controls		
Author	Total	AA	AC	CC	Total	AA	AC	CC	C	A	RAF	C	A	RAF
Hamon et al. [13]	103	55	36	12	168	84	66	18	60	146	0.71	102	234	0.70
Volzke et al. [14]	160	80	64	16	351	195	133	23	96	224	0.70	179	523	0.75
Hertwig et al. [15]	46	23	19	4	99	52	38	9	27	65	0.71	56	142	0.72
Wijpkema et al. [16]	324	150	130	44	2622	1271	1133	218	218	430	0.66	1569	3675	0.70
Li et al. [17]	307	116	100	91	176	100	55	21	282	332	0.54	97	255	0.72
Zhu et al. [18]	65	56	9	0	251	222	26	3	9	121	0.93	32	470	0.94
Zeng et al. [19]	54	45	9	0	371	310	59	2	9	99	0.92	63	679	0.92
Azova et al. [20]	52	25	17	10	59	34	19	6	37	67	0.64	31	87	0.74

A case-control design was used in all included studies. RAF = risk allele frequency; risk allele = C allele.

**Table 4:** Subgroup analyses of the association between *AGTR1* rs5186 polymorphism and restenosis.

Subgroup		Number	Odds Ratio	95% Confidential Interval	*p* Value	I^2^ (%)
Allele model						
Ethnicity	Caucasian	5	1.18	(1.03, 1.34)	0.02	0.0
	Asian	3	1.89	(1.48, 2.40)	<0.001	69
Sample Size	≥400	4	1.37	(1.21, 1.56)	<0.001	80
	<400	4	1.09	(0.84, 1.41)	0.52	0.0
PCI type	PTCA	3	1.11	(0.90, 1.38)	0.324	0.0
	Stent	5	1.40	(1.22, 1.60)	<0.001	74
Homozygote model						
Ethnicity	Caucasian	5	1.58	(1.19, 2.09)	0.002	0.0
	Asian	3	3.35	(1.99, 5.64)	<0.001	0.0
Sample Size	≥400	4	2.15	(1.64, 2.82)	<0.001	50
	<400	4	1.19	(0.68, 2.10)	0.55	0.0
PCI type	PTCA	3	1.30	(0.80, 2.11)	0.29	0
	Stent	5	2.21	(1.66, 2.93)	<0.001	37
Heterozygote model						
Ethnicity	Caucasian	5	1.01	(0.84, 1.22)	0.88	0.0
	Asian	3	1.42	(1.02, 1.98)	0.04	0.0
Sample Size	≥400	4	1.11	(0.93, 1.33)	0.26	20
	<400	4	1.04	(0.73, 1.47)	0.84	0.0
PCI type	PTCA	5	1.05	(0.79, 1.41)	0.74	0.0
	Stent	3	1.12	(0.92, 1.35)	0.27	0.0
Recessive model						
Ethnicity	Caucasian	5	1.59	(1.21, 2.08)	0.01	0.0
	Asian	3	2.89	(1.75, 4.78)	<0.001	0.0
Sample Size	≥400	4	2.02	(1.44, 2.84)	<0.001	25
	<400	4	1.23	(0.71, 2.14)	0.46	0.0
PCI type	PTCA	3	1.29	(0.81, 2.06)	0.29	0.0
	Stent	5	2.07	(1.53, 2.81)	<0.001	5.5
Dominant model						
Ethnicity	Caucasian	5	1.11	(0.94, 1.32)	0.23	0.0
	Asian	3	1.76	(1.30, 2.38)	<0.001	49
Sample Size	≥400	4	1.27	(1.09, 1.52)	<0.001	69
	<400	4	1.07	(0.77, 1.48)	0.69	0.0
PCI type	PTCA	3	1.10	(0.83, 1.44)	0.51	0.0
	Stent	5	1.24	(1.06, 1.44)	0.004	59

## Data Availability

The data used to support the findings of this study are available from the corresponding author upon request.
